# Effects of a refugee elective on medical student perceptions

**DOI:** 10.1186/1472-6920-9-15

**Published:** 2009-04-09

**Authors:** Kathleen Bronson Dussán, Erin M Galbraith, Mary Grzybowski, Bonnie Motyka Vautaw, Linda Murray, Kim A Eagle

**Affiliations:** 1 Michigan State College of Human Medicine, East Lansing, MI, USA; 2 Emory University Hospitals, Department of Cardiology, Atlanta, GA, USA; 3 Wayne State University School of Medicine, Department of Emergency Medicine, Detroit, MI, USA; 4 Ford Motor Credit Company, Dearborn, MI, USA; 5 University of Michigan, Department of Cardiology, Ann Arbor, MI, USA

## Abstract

**Background:**

There are growing numbers of refugees throughout the world. Refugee health is a relatively unstudied and rarely taught component of medical education. In response to this need, a Refugee Health Elective was begun. Medical student perceptions toward cultural aspects of medicine and refugee health before and after participation in the elective were measured.

**Methods:**

Preliminary questionnaires were given to all preclinical students at the academic year commencement with follow-up questionnaires at the refugee elective's conclusion. Both questionnaires examined students' comfort in interacting with patients and familiarity with refugee medical issues, alternative medical practices, and social hindrances to medical care. The preliminary answers served as a control and follow-up questionnaire data were separated into participant/non-participant categories. All preclinical medical students at two Midwestern medical schools were provided the opportunity to participate in the Refugee Health Elective and surveys. The 3 data groups were compared using unadjusted and adjusted analysis techniques with the Kruskall-Wallis, Bonferroni and ANCOVA adjustment. P-values < 0.05 were considered significant.

**Results:**

408 and 403 students filled out the preliminary and follow-up questionnaires, respectfully, 42 of whom participated in the elective. Students considering themselves minorities or multilingual were more likely to participate. Elective participants were more likely to be able to recognize the medical/mental health issues common to refugees, to feel comfortable interacting with foreign-born patients, and to identify cultural differences in understanding medical/mental health conditions, after adjusting for minority or multilingual status.

**Conclusion:**

As medical schools integrate a more multicultural curriculum, a Refugee Health Elective for preclinical students can enhance awareness and promote change in attitude toward medical/mental health issues common to refugees. This elective format offers tangible and effective avenues for these topics to be addressed.

## Background

The Geneva Convention defines refugees as "those persons who are forced to leave their countries of origin due to well-founded racial, religious, social, political persecution or of its possible persecutory threat [1-3]." Even with 3.2 million refugees and asylees resettling in the United States since 1975 [4], researchers are only beginning to investigate the effect refugees on have health care institutions, urgent care facilities and emergency departments. Refugees present with a higher frequency of challenging diseases, such as tuberculosis, hepatitis B, lead poisoning, parasitic infections, trauma, and mental illnesses [3]. Simultaneously, physicians are faced with the secondary challenge of the diverse health beliefs of refugees that don't always conform to Western norms. If physicians do not appreciate these socio-cultural differences, refugees seeking care are at risk of sub-optimal care, adverse health outcomes, and poor medical adherence [5-7].

Because of the proximity of our medical allopathic and osteopathic schools to over 11,000 refugees in our immediate community and 3,100 more residing in the outlying Mid-state area [8], our medical students identified a need for increased physician awareness of this growing population. In response to this need, an elective was developed and the overall objectives were to (1) develop a medical school course to address the medical needs of refugees by (a) educating medical students about refugee medical and mental health issues and cross-cultural care issues and (b) providing students with an opportunity to interact with refugees, and (2) measure their beliefs/attitudes about refugee medical care before and after the implementation of the elective. The discussion was done in the context of the global refugee phenomenon and its effects on both the developed and developing countries, not just the Western world, but with a focus on local refugee care. It was hoped that an increased knowledge base would provide a foundation for clinical interaction after the pre-clinical years. We studied medical student knowledge and attitudes toward refugee health care prior to and after implementing a newly-developed medical school elective directed at refugee health. To our knowledge, there have not been any studies investigating the change in medical student overall knowledge and attitudes pre- and post-elective toward refugees and their health compared to their non-participant peers' knowledge and attitudes.

## Methods

### Refugee Elective: Curriculum and Format

The Refugee Health Elective curriculum consisted of a course elective administered by both the College of Human Medicine and College of Osteopathic Medicine at Michigan State University. The elective was developed by a panel of faculty and students interested in the subject matter. Extensive research regarding the background and evolution of refugee awareness was conducted and reviewed for content relevant to optimizing the interpersonal communication and health care priorities set by *Physicians for Human Rights*, which addresses human rights, ethics, and advocacy issues addressing the health of refugee patients. Various professors, community physicians, and social agency workers, who serve as local experts in the field of refugee advocacy and care delivered lectures for the medical students. Students also had contact with refugees in the classroom setting.

All preclinical medical students enrolled in both colleges during the academic year 2005–2006 were eligible to participate in the elective. The course elective was conducted on one afternoon a week during the fall and early winter semesters of the study period. The course was an hour in length and included didactic and discussion sessions. Each class addressed specific topics relevant to refugee health, which are listed in table 1.

**Table 1:** Topics addressed in refugee health elective.

1.	Discussions on "A Global and Local Overview of Displaced Persons," a lecture prepared by Professor Barry N. Stein, Ph.D. of Michigan State University.
2.	State and local outreach agencies available to assist refugees.

3.	Psychological manifestations and assessment in the refugee population.

4.	Legal rights of refugees.

5.	Healthcare issues, common physical ailments and mental illnesses, and culturally appropriate care and cultural norms.

6.	The United Nations Guidelines for refugee health care and assessment.

7.	Sociologic perspectives of refugees and factors influencing refugees to flee their countries of origin.

8.	A refugee discussion panel with personal sharing from local refugees.

9.	A lecture on the international, medical situation for refugees with focus on the African refugee situation.

10.	A viewing of a documentary film *The Lost Boys of Sudan*, produced by Megan Mylan and Jon Shenk, followed by a critical discussion.

11.	Access to and discussion of the book, *The Middle of Everywhere *authored by Mary Pipher.

### Survey Data Collection

Data to evaluate the course were obtained from a baseline questionnaire administered to all first and second year students during mandatory medical school lectures or after scheduled examinations prior to the commencement of the course. Post-course questionnaires were available and administered to the same students regardless of course enrollment following the elective's conclusion. Non-participants were asked to complete the post-elective questionnaire to serve as a control group. Neither questionnaire was mandatory.

This study was approved by the Human Investigative Committees (HIC) at Michigan State University. However, the HIC prohibited collection of demographic variables such as, student identification number or study number linking student participation in the pre- and post-elective questionnaires, as well other demographic variables.

### Data Collection Instrument

The questions on the pre- and post-elective questionnaires comprised 20 items and were identical except for three questions on the post-elective survey regarding student-refugee interactions that occurred during the course of the elective. Demographic variables (yes|no) included whether the student was multilingual, considered themselves a minority, or had at least one previous interaction with a refugee. Fourteen items reflecting attitudes and beliefs of the cultural domains of practicing medicine were also included. The students were asked to quantify the degree to which they agreed or disagreed to each item using a 5-point Likert scale. The fourteen attitudinal and belief items included: personal comfort interacting with and obtaining personal and medical histories from foreign-born patients as well as patients who experienced rape, torture or violence; knowledge of medical and mental health issues relevant to refugees; the ability to identify cultural differences in traditional and alternative medical care practices; self-perceived understanding of logistical and cultural/social obstacles of medical compliance relative to refugees; and lastly, the degree to which one's own race, gender, and religion would impact his/her ability to practice medicine. The follow-up questionnaire contained four additional questions (yes|no) regarding if and how the elective benefited the students.

### Statistical Analyses

Questionnaire data were classified into three groups: students completing the questionnaire prior to the elective (the baseline or control group), students who enrolled in the elective, and students who did not enroll in the elective. Because we could not assign study identification numbers to the questionnaires, we were unable to directly compare individual pre- and post-questionnaires. The three data groups were therefore treated as independent entities.

Odds ratios and 95% confidence intervals were calculated to determine the degree to each variable was associated with participation status. For dichotomous responses, chi-square tests were used to determine frequency differences between the groups. Likert scale responses were scored as follows: 5 = strongly agree, 4 = agree, 3 = neutral, 2 = disagree, and 1 = strongly disagree, and these data were treated as continuous variables. Means and standard deviations were calculated for each item. The Shapiro-Wilks test was used to determine if the Lickert scale responses were normality distributed, and the responses were not normally distributed. The Wilcoxon Rank Sum test was used to determine which treatment group means differed when the Kruskall-Wallis test was significant. In order to correct for the multiple comparisons between treatment groups, Bonferroni correction was applied. An analysis of covariance (ANCOVA) was used to adjust for demographic variables that had significant associations with the treatment groups. All reported *p*-values are two-tailed. A *p*-value less than or equal to 0.05 was considered a statistically significant. All analyses were performed using SAS version 9.1.3 [9].

## Results

### Subjects

The first and second year medical school classes of both College of Human Medicine (CHM) and College of Osteopathic Medicine (COM) consisted of a total of 556 students. Of these, 408 (73.4%) responded to the baseline questionnaire and 403 (72.5%) to the follow-up questionnaire. A total of 43 (7.7%) students enrolled in the elective with 42 of the 403 respondents (10.4%) to the follow-up questionnaire having participated in the elective.

Minority students were more likely to enroll in the Refugee Health Elective as seen in the follow-up questionnaire that showed 46.3% of participants versus 25.6% of non-participants considered themselves minorities, (p = 0.02). Similarly, elective participants were more frequently multilingual (69.1% of participants versus 43.0% of non-participants; p < 0.01). See Table 2.

**Table 2:** Demographic characteristics of first and second year medical school students eligible to enroll in the refugee elective

		**Participation in Refugee Elective**		
	**Baseline (n = 408)**	**Yes (n = 42)**	**No (n = 361)**	
**Characteristic**	**N (%)**	**N (%)**	**N (%)**	***p*-value**
Considers oneself a Minority	107/408 (26.3)	19/41 (46.3)	92/359 (25.6)	0.0161
Multi-lingual	201/408 (49.3)	29/42 (69.1)	154/358 (43.0)	0.0037

### Perceptions

Students who participated in the elective expressed a deeper understanding of the various cultural influences on patient care when compared to the baseline group and those not completing the elective (Table 3).

**Table 3:** Belief/attitude responses: differences among three groups: baseline, participants, and nonparticipants

			Enrollment Status		P-values for Overall and Multiple Comparisons for Between Group Differences Given One Significant Mean Difference Between Three Groups (BL = Baseline ENRL = Enrolled NE = Not enrolled)				
		
Item	Belief/Attitude	Baseline (n = 408) mean (SD)	Yes (n = 42) mean (SD)	No (n = 361) mean (SD)		p-value	BL vs ENRL	BL vs NE	ENRL vs NE
1	I am comfortable interacting with patients	4.48 (0.60)	4.50 (0.72)	4.47 (0.57)	Unadjusted	.7040	---	---	---
					Adjusted ^§^	.6688	---	---	---
2.	I am comfortable interacting with foreign-born patients.	4.36 (0.64)	4.52 (0.77)	4.28 (0.59)	Unadjusted	.0017 *	.1865	.1282	.0079*
					Adjusted ^§^	.0023 *	.2359	.5970	.0323*
3.	I am comfortable obtaining a personal and medical history from patients.	4.38 (0.63)	4.41 (0.71)	4.38 (0.60)	Unadjusted	.7386	---	---	---
					Adjusted ^§^	.9280	---	---	---
4.	I am comfortable obtaining a personal history from foreign-born patients.	4.25 (0.73)	4.41 (0.50)	4.17 (0.72)	Unadjusted	.0646	---	---	---
					Adjusted ^§^	.0606	---	---	---
5.	I am comfortable taking a sensitive torture/rape/violence history from a patient.	3.62 (0.99)	3.59 (1.00)	3.51 (1.01)	Unadjusted	.3880	---	---	---
					Adjusted ^§^	.3844	---	---	---
6.	I know the medical issues common to refugees.	2.21 (0.84)	4.05 (0.58)	2.21 (0.73)	Unadjusted	< .0001*	< .0001*	1.000	< .0001*
					Adjusted ^§^	< .0001*	< .0001*	1.000	< .0001*
7.	I know the mental health issues common to refugees.	2.15 (0.79)	4.05 (0.49)	2.21 (0.78)	Unadjusted	< .0001*	< .0001*	1.000	< .0001*
					Adjusted ^§^	< .0001*	< .0001*	1.000	< .0001*
8.	I am able to identify cultural differences in understanding medical/mental health conditions	3.24 (0.96)	4.00 (0.58)	3.45 (0.90)	Unadjusted	< .0001*	< .0001*	.0189*	.0010*
					Adjusted ^§^	< .0001*	< .0001*	.0069*	.0125*
9.	I am aware of and can identify alternative/traditional medical practices	3.35 (0.91)	3.86 (0.52)	3.44 (0.89)	Unadjusted	.0024*	.0035*	1.000	.0282*
					Adjusted ^§^	.0022*	.0130*	.8094	.1011
10.	I understand the logistical hindrances to medical follow-up and compliance	3.69 (0.80)	4.12 (0.55)	3.91 (0.71)	Unadjusted	< .0001*	< .0001*	.0189*	.0010*
					Adjusted ^§^	< .0001*	< .0001*	.0069*	.0125*
11.	I understand the cultural/social hindrances to medical follow-up and compliance.	3.63 (0.84)	4.14 (0.61)	3.89 (0.70)	Unadjusted	< .0001*	.0004*	.0002*	.0962
					Adjusted ^§^	< .0001*	.0016*	.0001*	.5888
12.	My race/ethnic background will not/does not affect my interactions with patients.	3.27 (1.24)	2.57 (1.15)	2.96 (1.20)	Unadjusted	< .0001*	.0028*	.0027*	.2088
					Adjusted ^§^	< .0001*	.0100*	.0012*	.8173
13.	My religious background will not/does not affect my interactions with patients.	3.51 (1.20)	2.90 (1.23)	3.26 (1.20)	Unadjusted	.0008*	.0164*	.0267*	.4297
					Adjusted ^§^	.0007*	.0213*	.0299*	.5719
14.	My gender will not affect my interactions with patients.	3.24 (1.21)	2.57 (1.15)	2.94 (1.14)	Unadjusted	< .0001*	.0041*	.0031*	.1984
					Adjusted ^§^	< .0001*	.0126*	.0025*	.7839

Abbreviations: SD, standard deviation; BL, baseline; PRT, participants; NP, nonparticipants; Vs, versus.

^§ ^Adjusted for self-reported minority status and multi-lingual status.

* Statistically significant

Those who enrolled in the elective demonstrated more comfort interacting with foreign-born patients (unadjusted (U) p =< 0.01; adjusted (A) p = 0.03). There was also a trend toward significance with students who participated in the elective feeling more at ease taking a personal and medical history from foreign-born patients than elective non-participants (U, A p = 0.06). Compared to baseline and to the non-elective students, elective participants showed significant change in their overall knowledge of refugee medical and mental health issues (U, A p =< 0.01) and in their ability to identify cultural differences in understanding medical/mental health conditions (U, A p = 0.01). Elective participants also gained the ability to understand logistical and cultural/social hindrances to medical follow-up/compliance. The difference between the means was significant for both the preliminary and the elective participant groups and for the preliminary and non-participant groups (U, A p < 0.01 for both). There was no statistically significant difference between the means for the participant and non-participant groups, however.

When asked to respond to three statements pertaining to the effect of their own personal identity of background (i.e. race/ethnicity, religion, and gender) on patient interactions, the means for the preliminary responses ranged from 3.24 to 3.51, indicating that at baseline the group tended to agree that their race, religion, and gender would not affect their interactions with patients. Data from the follow-up questionnaires revealed that elective participant mean answers ranged from 2.57 to 2.90, indicating that they as a group tended to disagree with the premise that their personal identifiers would not affect their interactions with patients, i.e. they felt that it would have an effect. For all three groups of responses, there was a perception that gender, followed closely by race, would have the most affect, i.e. the lowest means, indicating more disagreement with the statements, and that religion would have the least impact. When mean responses were compared, there were significant differences, (p < 0.05), between the preliminary and participant means as well as between the preliminary and the non-participant means, (Table 3).

Among elective participants, 41 of 42 (97.6%) indicated that they anticipated working with refugees in the future and 37 of 42 (88%) elective participants felt that participation in the elective prepared them clinically for the 3^rd ^and 4^th ^years of medical school. Of note, 5 of the 42 participant responders did not answer to this question. See Table 4 for results. Students were also given a venue on the survey to share their comments and feedback of the elective. Their feedback is listed in Additional file 1.

**Table 4:** Follow-up questionnaire responses to questions relative to the refugee elective among students who completed the elective (n = 42)

**Question**	**N (%)**
Was this your first interaction with non-simulated patients?	2/42 (4.8)
Was this your first clinical interaction with resident or attending physicians?	2/41 (4.9)
Do you feel that your experiences helped prepare you for 3^rd ^and 4^th ^year clinically?	37/42 (88.1)
Do you anticipate working with refugees in the future?	41/42 (97.6)

## Discussion

The expanding international refugee phenomenon is producing a growing need for physicians well-versed in the health problems of refugees. Various initiatives have been implemented throughout the world, including the countries of Great Britain, Canada, Australia, New Zealand [10,11] and the United States, to raise awareness of the culturally diverse societies in these countries [12]. Some medical schools have addressed multicultural issues with cultural immersion programs [13-16], international electives, discussions, and formal pre-clinical cultural competence seminars in which students discuss racial, religious, and sexual diversity in society [11,17-22]. But unlike cultural diversity discussions, refugee health has been much less studied and is a rarely taught component of medical education. Few medical students and physicians know how to appropriately treat refugees [4] or identify those who have undergone torture or related trauma [1,23-25]. Physicians untrained in issues sensitive to refugees and cultural diversity can have a deleterious effect on refugees. Prior research found that ineffective interactions with refugees resulted in longer office visits, delays in obtaining consent, unnecessary testing, and patient non-adherence. Medical residents also found it difficult to assess the degree of patient understanding of their illnesses and felt less prepared to care for those who hold different beliefs. Conversely, other reports have shown that many immigrants and refugees reported feeling misunderstood by their physicians [26]. Poor communication often exists between physicians and refugees, and patient satisfaction, adherence, and health outcomes have been tied to communication [6].

In a similar elective, Griswold described a unique 'Refugee Health Night' applying both academic seminars and clinical encounters for medical students at the State University of New York, Buffalo in 2003 [2]. Based on self-reported feedback, these students had an overall positive impression of their experience [2]. Griswold found a change in medical student attitudes towards cultural awareness, increased communication, and increased sensitivity to religious values, family patterns, and gender roles both before and after their interactions with the refugees [27,28].

We successfully developed and implemented a medical school elective course to expose the medical needs of refugees by 1) educating medical students about refugee medical and mental health issues, as well as cultural diversity issues and 2) providing students with an opportunity to interact with refugees. Through our questionnaires, we showed that the elective increased student comfort interacting with foreign-born patients and their knowledge of refugee medical and mental health issues. It also improved understanding of cultural differences in medicine and alternative/traditional medical practices, and increased awareness of the logistical, cultural, and social hindrances to medical care and compliance. Lastly, we showed that medical students acknowledged that their race, religious background, and gender may have an effect on their interaction with patients after learning about refugees' perceptions of medical care personnel.

Participating students were more likely to feel that their personal religion, ethnicity/race, and gender would affect their interactions with patients. This suggests that learning about refugees and cultural diversity led to an increased recognition of these personal factors. Previous research has shown that gender concordance results in more effective communication, respect, and trust [27,29,30]. Similarly, racial concordance can improve satisfaction with the delivery of health care services and shared-decision making [29]. While one cannot change their gender or race for each given patient interaction, recognition of the impact of these identifiers on the doctor-patient relationship is essential.

Our results are congruent with other projects aimed at multi-cultural medical education [13-16,18,27,31-33], and more specifically, health care for refugees [27,28]. These studies support the proposition that student attitudes about refugees change following coursework or clinical encounters with refugees.

The overwhelming majority of students reported that this elective helped prepare them for their last two years of medical school and that they anticipated working with refugees in the future. This reflects other studies which have found that long-term medical education programs can have a positive effect on student motivation to work with the underserved [34].

The primary advantage of our intervention study is that we assessed the same cohort of students prior to and following the course elective. To thoroughly consider what may have affected our findings, the role of self-selection bias must be addressed because this course was not mandatory. Those who enrolled in the elective may have been initially more interested in refugee health issues or have had prior experiences with refugees thereby making them more inclined to enroll in the elective. We also adjusted for student race and multilingual abilities. For example, those who enrolled in the elective felt more able to identify alternative and traditional medical practices. Following adjustment for race and multilingual status, this difference was no longer significant. The lack of significance following adjustment, despite that medical practices were addressed in the course, suggests that these particular student-held-attitudes may have been inherent among students with multi-racial backgrounds and those who were multilingual.

It is also plausible that the experience of being a medical student, regardless of enrollment status, during the study period accounts for some of the non-significant findings between those who did and did not enroll in the elective. For example, there was no statistical significance found between those who did and did not enroll in the elective regarding understanding cultural and social hindrances to medical follow-up and compliance. This finding suggests that knowledge was likely acquired through either other medical school courses or experiences resulting in an overall greater awareness of these factors over time.

Although this was a preliminary investigation as to how an elective focused on refugees would influence medical students' knowledge and perceptions, there are several disadvantages inherent in this study. The primary disadvantage is that we were unable to assign study identification numbers to link pre- and post-study questionnaires. Secondly, we did not obtain other demographic variables (age, gender, year of medical schooling, religion, future medical specialty interest). In our future investigations, we will incorporate more demographic variables so that we can determine which potentially confounding personal factors are associated with enrolling in a Refugee Health Elective. As this was the first time the Refugee Health elective was offered, our sample size (42 students) was limited; therefore, we were at a disadvantage to measure all the student differences and to precisely estimate the degree to which attitudes changed during the course of the semester. Additional analyses as the elective continues to evolve would give the project additional validity. Since 41 of the 42 (97.6%) participants indicated a desire to work clinically with refugees in the future, a long-term study analyzing the career paths of these future physicians would demonstrate the long-standing effects of this and similar elective courses. In addition, we believe that incorporating other health care professionals, such registered nurses, in the elective would have provided the students with additional perspectives especially as nurses are often the mediators of the patient-physician interaction process.

In terms of the elective course curriculum, and the limited contact with refugees, we would have also have liked to have included additional clinical experiences. However, in our situation, sensitivity and privacy issues led to limited established avenues by which students could participate in delivering medical and mental health care to refugees.

## Conclusion

Refugees are among the world's most vulnerable inhabitants and their numbers grow annually. With a growing increase in refugees worldwide, physicians will be more likely to find themselves having to provide care for this subset of the population [26]. Our study provides evidence that a medical elective focused on refugee health can provide an increased knowledge base and change in attitude of medical students toward refugee health and care. This elective format offers a tangible and effective avenue for these topics to be addressed for future medical clinicians. We must make our future physicians aware of this unmet and misunderstood medical need. Focused research relative to issues important to refugees and required characteristics of physicians to create successful interactions with refugees is paramount.

## Competing interests

Regarding the full disclosure of any potential conflict of interest, Dr. Eagle has research grants from the following: Biosite, BristolMyers Squibb, Cardiac Sciences, Blue Cross Blue Shield of Michigan, Hewlett Foundation, Mardigian Fund, Pfizer, Sanofiaventis, and the Varbedian Fund. He is also is a Consultant/Advisory Board for the following: NIH, NHLBI, Pfizer, Sanofiaventis, and the Robert Wood Johnson Foundation. The other authors of the manuscript "Effects of a Refugee Elective on Medical Student Perceptions" do not have any competing conflict of interests (financial, political, personal, religious, academic, ideological, commercial or any other) to report nor have any ties to for-profit companies or institutions.

## Authors' contributions

KBD assisted in developing and executing the elective, designing the survey tool, and writing and editing the primary manuscript. EG assisted in developing the survey tool and assisted in both writing and editing the primary manuscript. MG did the statistical analysis and contributed in editing the manuscript. BMV assisted with the statistical analysis and the editing of the manuscript's methods section. LM helped to develop and execute the elective and edit the manuscript. KE assisted in the survey design, funding, and in the editing of the manuscript and overall guidance of the elective.

## Pre-publication history

The pre-publication history for this paper can be accessed here:



## Supplementary Material

Additional file 1 **Comments from Elective Participants.** Click here for file
