# Supplementary material for: Effects of a refugee elective on medical student perceptions
Source: BMC Med Educ. 2009 Apr 9;9:15. doi: 10.1186/1472-6920-9-15 (PMC2676284; doi:10.1186/1472-6920-9-15)
Supplement: Additional file 1 — Comments from Elective Participants. [file 1472-6920-9-15-S1.doc]

Elective participants shared various comments on their experiences from this

elective. The comments were as follows:

• “[I] am more aware of refugee health issues, especially mental health (ie,

PTSD). I am also more aware of the local resources for refugees. I am more

confident in my ability to interact with refugee patients and take a history,

even if it involves violent [history];”

• “I understand issues and barriers more completely;”

• “[The elective] made me aware of situations and it shed light on the troubles

and ordeals that refugees face;”

• “It opened my eyes to a new population of patients and issues I will see in

clinicals;”

• “It made me more aware of possible situations I may encounter with patients;”

• “Fascinating class. Very informative;”

• “[I have] a greater awareness of refugee issues…many issued refugees face

that I was not aware of;”

• “…it gave me a more clear picture of the unique history of each refugee

patient;”

• “I am much more aware of the historical background behind situations that

lead to creation of refugees and internally displaced persons (IDPs). I am

more aware of their mental, physical, emotional, and social struggles. I will

be better prepared to provide patient-centered care for a refugee;”

• “I was completely unaware of some of the issues of refugee health;”

• “[it] helped broaden my understanding of refugee health issues and

background;”

• “I learned a lot about what issues refugees are facing and what their needs

are;”

• “It was enlightening;”

• “[It gave me] a better grasp of health and mental health concerns. I didn’t

even know the full definition of a refugee before this elective;”

• “I learned so much about refugees and their care/health issues. More students

should take this class.”
